# Repeat Breast Ultrasound Demonstrates Utility with Added Cancer Detection in Patients following Breast Imaging Second Opinion Recommendations

**DOI:** 10.1155/2022/1561455

**Published:** 2022-01-31

**Authors:** R. Jared Weinfurtner, Melissa Anne Mallory, Dayana Bermudez

**Affiliations:** Moffitt Cancer Center, 10920 McKinley Dr., Tampa, FL 33612, USA

## Abstract

**Purpose:**

Second opinion consultation for patients with suspicious findings on breast imaging and patients with known breast cancer is not uncommon. We sought to determine the frequency of second opinion breast and axillary ultrasound imaging review and the subsequent impact on clinical management.

**Materials and Methods:**

An IRB-approved retrospective chart review was conducted on 400 consecutive patients with second opinion radiology interpretations performed by subspecialized breast radiologists at a designated cancer center, including mammogram and ultrasound review. The outside institution imaging reports were compared with second opinion reports to categorize ultrasound review discrepancies which were defined as any BI-RADS category change. The discrepancy frequency, relevant alterations in patient management, and added cancer detection were measured.

**Results:**

The second opinion imaging review resulted in discrepant findings in 108/400 patients (27%). Patients with heterogeneously or extremely dense breasts had higher discrepancy frequency (36% discrepancy, 68/187) than those with almost entirely fatty or scattered fibroglandular breast tissue (19% discrepancy, 40/213) with *P* = 0.0001. Discrepancies resulted in the following changes in impression/recommendations: 70 repeat ultrasounds for better characterization of a breast lesion, 11 repeat ultrasounds of a negative region, 20 repeat ultrasounds for benign axillary lymph nodes, 5 downgrades from probably benign to benign, and 2 upgrades from benign to suspicious. Repeat ultrasounds of the axilla in 19 patients resulted in 13 biopsy recommendations, and 4 were metastatic (PPV3 31%). In the breast, repeat ultrasounds in 81 patients resulted in 14 upgrades to suspicious. Of these, 5 yielded malignancy. In addition, one patient was upgraded from benign to suspicious based on the outside image, with pathology revealing malignancy (breast PPV3 40%). Breast lesion BI-RADS category downgrades in 27 patients resulted in 10 avoided biopsies. Ultimately, second opinion ultrasound review resulted in altered management in 12% of patients (47/400). This included discovery of additional breast malignancies in 6 patients, metastatic lymph nodes in 4 patients, excisional biopsy for atypia in 1 patient, 4 patients proceeding to mastectomy, 10 patients who avoided biopsies, and 22 patients who avoided follow-up of benign findings.

**Conclusions:**

In this study, subspecialized second opinion ultrasound review had an impact on preventing unnecessary procedures and follow-up exams in 8% of patients while detecting additional cancer in 2.5%.

## 1. Introduction

Tertiary and dedicated care centers utilize a multidisciplinary approach in the evaluation and treatment of cancer patients. With increasing frequency, patients present to these institutions with radiologic and pathologic examinations performed elsewhere leading to request for second opinion review of those studies [1]. Second opinion interpretation of imaging by subspecialized radiologists has been previously shown to improve diagnostic ability and alter patient management [2–8]. In particular, breast fellowship-trained radiologists demonstrate increased cancer detection compared to their general counterparts [8].

A recent study by Whorms et al., for instance, focused on assessing the incidence and clinical significance of discrepancy in subspecialty interpretation of outside breast imaging at a tertiary cancer center [9]. They found a discrepancy rate of 16% which resulted in a change in management in 7% of all cases and detection of additional malignancies in 4%. There are, however, scant data on the specific impact of breast and axillary ultrasound second opinion imaging review. In a recent study of 209 patients with second opinion breast ultrasound review, clinical management was altered in 33%, with additional cancer detected in 5% of patients [10]. The purpose of our study was to determine breast ultrasound review discrepancy frequency and the subsequent utility and impact on patient management in a large cohort of patients pursuing breast imaging second opinion.

## 2. Methods

**Figure 1:**
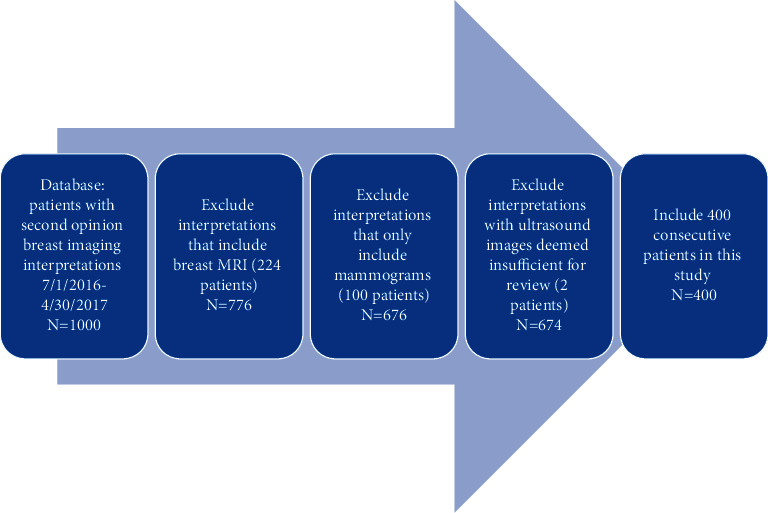
Inclusion and exclusion criteria leading to the inclusion of 400 consecutive patients with second opinion breast ultrasound review.

After receiving institutional review board approval with waiver of informed consent, a retrospective chart review was performed on a database of 1000 consecutive patients presenting for second opinion breast imaging review to a single National Comprehensive Cancer Network (NCCN) designated cancer center from July 1, 2016, through April 30, 2017. Review of prior second opinion breast imaging studies demonstrated sample sizes ranging 149–380 [1, 2, 4–8, 11]. Therefore, 400 consecutive patients from this database that included mammogram and ultrasound review, without MRI review, were included (Figure 1). As results from this database on breast MRI second opinion review have been published previously [4] and to focus on ultrasound review specifically, interpretations that included MRI review were excluded from this study. In addition, patients were excluded if their outside ultrasound images were deemed of insufficient quality for review by the interpreting second opinion radiologist.

Patients presenting for breast care at our institution received an interpretation of outside imaging by one of eight breast fellowship-trained radiologists with between one and twelve years of experience. A minority of cases were read first by the breast imaging fellow-in-training. The process includes reinterpretation of the patient's most recent mammograms obtained within the preceding six months from the day of interpretation. If the patient also has breast ultrasound, axillary ultrasound, and/or breast MRI studies performed within the previous six months, review of those studies is also performed (noting limitations due to differences in acquisition, hardware/software, and operator-dependent image acquisition at the time of the report). All outside breast and axillary ultrasounds are loaded into our Patient Archiving and Communication System (centricity by General Electric Healthcare, Boston, Massachusetts) by our dedicated breast imaging coordinator and reviewed on a dedicated workstation. Cases of insufficient quality as determined by the interpreting radiologist are not included in the formal second opinion report and stated as such. For each patient, the attending radiologist issues a single report containing his or her findings and recommendations, including the overall BI-RADS assessment and need for biopsy or additional imaging. Clinical evaluation by the clinician(s) and reinterpretation of the outside institution pathology are performed by corresponding members of the multidisciplinary team in conjunction with second opinion imaging interpretation.

The outside institution imaging reports were compared with second opinion reports to categorize ultrasound review discrepancies. These were defined as any Breast Imaging Reporting and Data System (BI-RADS) category change for any of the noted findings mentioned in the ultrasound section of the report. For patients with more than one discrepancy, outcome categorization was made based on the overall final BI-RADS category in order to analyze results on a per-patient basis. Patients were categorized as having altered clinical/surgical management based on ultrasound review discrepancy for several outcomes. This included detection of additional malignancy, detection of additional high-risk lesion(s) for which surgical excision was recommended, BI-RADS category downgrades (BI-RADS 4 or 5 downgraded to 1, 2, or 3 and BI-RADS 3 downgraded to 1 or 2), and patients proceeding to mastectomy after recommendation for additional biopsies. For patients with BI-RADS category downgrades, the findings were evaluated for 3-year stability.

The discrepancy frequency, relevant alterations in patient management, and added cancer detection were calculated. In addition, histologic reports, clinical and surgical reports, and demographic data were extracted for complete review. Patient factors including breast density, availability of prior studies, and initial diagnosis at time of presentation for second opinion review were also collected and evaluated for association with discrepancy frequency. This was evaluated statistically using Fisher's exact test with a *P* value <0.05 considered statistically significant. Calculations were performed using Social Science Statistics software (https://www.socscistatistics.com).

## 3. Results

A total of 400 consecutive patients were evaluated. Patients ranged in age from 26 to 90 years (median age: 59 years), and all but one were female. Outside institutions were primarily private/community practices (393/400, 98%) with only a minority coming from academic institutions (7/400, 2%). Only a small minority of second opinion interpretations were first read by the fellow-in-training (4%, 17/400). The majority of patients presented with a tissue diagnosis of invasive or in situ breast malignancy (58%, 231/400) with additional indications listed in Table 1.

**Figure 2:**
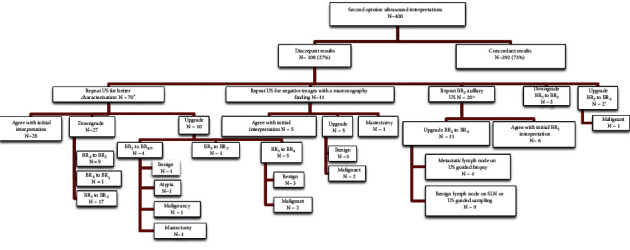
Flowchart analysis displaying the results for patients with second opinion breast imaging ultrasound review. “*N*” = number of patients. ^*∗*^Three patients lost to follow-up and two proceeded to mastectomy. ^One patient lost to follow-up. ‘One patient lost to follow-up.

**Figure 3:**
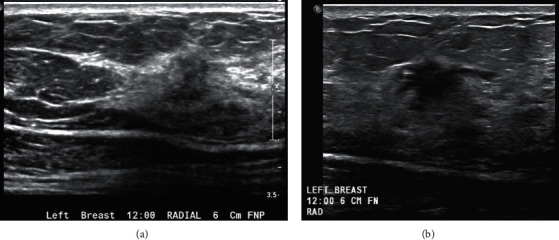
A 60-year-old female presented for second opinion breast imaging interpretation after the percutaneous ultrasound-guided biopsy of a left axillary lymph node revealed metastatic disease favoring primary breast cancer. Second opinion review included bilateral 2-dimensional digital mammography screening study and a bilateral handheld whole breast ultrasound screening study performed after the screening mammogram was read as normal with heterogeneously dense breasts. The outside report recommended ultrasound biopsy for suspiciously enlarged left axillary lymph nodes. The breasts were read as negative in the outside report. On second opinion interpretation of the sonographic images, the radiologist noted a hypoechoic lesion depicted at 12:00 6 cm from the nipple in the left breast (a). Repeat ultrasound for better characterization was performed and depicted an irregular hypoechoic mass spanning 19 mm with angular margins (b). Ultrasound-guided biopsy was recommended and revealed invasive ductal carcinoma.

Discrepant second opinion breast and axillary ultrasound review was seen in 108/400 patients (27%). The leading reason for discrepancy was repeat ultrasound for better characterization of a breast lesion in 70 patients. Additional discrepancies included 11 repeat ultrasounds of a negative region, 20 repeat ultrasounds for benign axillary lymph nodes, 5 downgrades from BI-RADS 3 to BI-RADS 2, and 2 upgrades from BI-RADS 2 to BI-RADS 4 (see Figure 2). In the axilla, repeat ultrasounds in 13/19 patients (68%) resulted in upgrade to biopsy recommendations. Of these, 4/13 yielded metastatic nodal disease for positive predictive value 3 (PPV3) of 31%. In the breast, 14/81 repeat ultrasounds (17%) led to recommendation for biopsy which yielded 5/14 additional malignancies (PPV3 36%). One additional patient was upgraded from a BI-RADS 2 to a BI-RADS 4 category based on the provided outside images with pathology revealing malignancy (see Figure 3). In addition, 27 patients were downgraded after repeat ultrasound, resulting in ten avoided biopsies. All patients downgraded to a BI-RADS 2 or 3 that continued their care at our institution demonstrated no interval malignancies at the site of interest after three years. Overall, discrepant second opinion breast ultrasound review resulted in added or avoided biopsies in 38/108 patients (35%).

Patients were more likely to have discrepant ultrasound interpretations if they presented with heterogeneously or extremely dense breasts (36% discrepancy, 68/187) compared to those with almost entirely fatty or scattered fibroglandular breast tissue (19% discrepancy, 40/213) with *P* = 0.0001. Presentation with a current diagnosis of invasive or in situ breast malignancy was associated with lower discrepancy frequency (15%, 34/231) than other presentations (44%, 74/169). Patients were also less likely to have second opinion review resulting in a recommendation for an additional ultrasound biopsy, specifically, if they presented with a current diagnosis of breast malignancy (9/231 (4%) vs 16/169 (9%) with *P* = 0.035). Availability of any prior breast imaging for comparison was not associated with a significant difference in discrepancy frequency compared to those without (see Table 2).

Ultimately, second opinion ultrasound review recommendations resulted in altered management in 12% of patients (47/400). This included diagnosis of additional breast malignancies in 6 patients, metastatic lymph nodes in 4 patients, excisional biopsy for atypia in 1 patient, 4 patients proceeding directly to mastectomy, 10 patients avoiding further biopsy, and 22 patients avoiding further follow-up of benign findings.

## 4. Discussion

While prior studies have demonstrated the added value of second opinion imaging interpretations in regard to additional cancer detection and relevant changes in management, there are limited data on the specific impact of breast ultrasound second opinion imaging review on the management of breast cancer patients [1–3, 5, 10]. In this study, second opinion review of outside ultrasound imaging resulted in discrepant findings in 27% of patients, leading to the detection of additional cancer in 10 patients (2.5%) and changes in clinical management in 12%. This adds to the growing body of literature supporting the value of subspecialized imaging interpretation in the management of the patients undergoing workup and management of suspicious breast imaging findings, including cancer.

For the role of ultrasound specifically, our study demonstrates that specialized second opinion review of outside breast and axillary ultrasounds is of clinical utility. This is in spite of the possible limitations associated with retrospectively reviewing the operator-dependent images. Similar to our study, Horvat et al. evaluated additional biopsies performed or averted by second-look ultrasound resulting from second opinion review in a cohort of 209 patients. In their study, 33% of patients with second-look ultrasound resulting from discrepant review resulted in additional biopsies being performed or averted, which is similar to the 35% observed in our study. They also demonstrated a PPV3 of 25% for the added biopsies, slightly lower than the PPV3 of 36% in the breast and 31% in the axilla demonstrated in our study [10].

A study by Song et al. in 2015 found that the two most common reasons for missed actionable findings on ultrasound examinations were misinterpretation of a suspicious feature and multiple distracting lesions [12]. This is supported in our results with added biopsy recommendations for 10/65 (15%) patients with repeat breast ultrasound for better characterization and 13/20 (65%) patients with repeat axillary ultrasounds. In addition, one patient initially categorized as a BI-RADS 2 on the outside report was upgraded to BI-RADS 4 based on second opinion review, and that biopsy resulted in malignant pathology.

The 27% discrepancy frequency in our study is similar to that for previous studies of second opinion breast imaging review which ranges from 18 to 57% [1–8, 10]. A previous study focusing specifically on MRI discrepancy frequency demonstrated a lower frequency (18%) than breast imaging review overall. This is expected, given that other studies evaluated discrepancies related to mammogram, ultrasound, and MRI combined. Thus, our discrepancy frequency of 27% suggests that ultrasound review may contribute to a higher proportion of overall discrepancies than does MRI review. The added cancer detection in our study (2.5%) is also similar to those previous studies with a range of 2–5% as is the impact on overall patient clinical/surgical management (12% in our study vs 10–27% in prior studies) [1–3, 5, 7, 10].

In terms of associations with breast ultrasound review discrepancy, we demonstrated a higher discrepancy frequency for patients with dense breasts. This result matches that of a previous study of second opinion breast imaging review [3]. Given that the presence of dense breasts is an independent risk factor for breast cancer and that ultrasound has higher sensitivity than mammogram alone in patients with dense breasts, it is perhaps appropriate that those patients are more likely to have recommendations for additional ultrasound imaging [13, 14]. We also demonstrated a decreased discrepancy frequency in patients presenting with a diagnosis of breast cancer compared to other presentations. This likely relates to the fact that these patients are further along in their workup (e.g., after biopsy has been performed) compared to those without a tissue diagnosis. Indeed, ultrasound discrepancies were more than twice as likely to result in an additional ultrasound biopsy recommendation in patients without a current diagnosis of malignancy. Finally, while previous studies have shown lack of prior comparison imaging as a risk factor for increased discrepancy frequency, this was not the case in our study [3, 4]. This likely relates to the higher reliance on prior mammograms for comparison, as they cover the entire breast [15]. Targeted ultrasound, in contrast, is less likely to have imaged a specific target previously and is, therefore, not often useful for comparison.

Limitations of our study are similar to comparison studies of second opinion breast imaging. First, our study is limited by the variability in experience of the outside sonographers as well as the outside interpreting radiologists. Thus, added cancer detection and management changes may not be solely attributed to breast radiology specialists but could be attributed to the effect of double reading. Indeed, double reading of mammogram studies results in increased sensitivity at the expense of higher recall rates [16–20]. However, the impact of double reading on ultrasound is less clear, with most studies involving automated whole breast ultrasound [21, 22]. In addition, the influence of mammogram reinterpretation inevitably played a role in some ultrasound discrepancies, particularly for the patients with recommendations for repeat ultrasound of a negative region. Presumably, the interpreting radiologist was looking for a correlate for a mammographic finding in these cases. However, the role of the mammographic appearance on the other discrepancy categories is not as easily assessed. Next, this retrospective study did not have a control group for outcome comparison. For instance, four patients with recommendations for additional ultrasound imaging or biopsy instead proceeded to mastectomy. It is unclear if the patient would have pursued this route without second opinion interpretation and if mastectomy was the optimal long-term treatment for these patients. Future prospective studies may overcome these limitations and further define the impact of second opinion breast ultrasound review. Finally, patients with breast MRI review were excluded from our study, and thus, the impact of breast MRI review on ultrasound discrepancy was not evaluated. However, data on breast MRI review were published previously [4] and breast MRI review is more likely to artificially increase ultrasound discrepancy frequency due to the higher sensitivity of MRI for the detection of breast cancer [23].

In conclusion, second opinion breast and axillary ultrasound resulted in the detection of additional cancer in 2.5% of patients, downgrades in 8%, and overall change in management for 12%. This resulted from a discrepancy frequency of 27% and subsequent recommendations for additional tissue diagnosis in 7% of patients (with PPV3 of 36%). These findings demonstrate added clinical/surgical impact for specialized second opinion breast ultrasound review.

## Figures and Tables

**Table 1:** Initial diagnosis on presentation for patients presenting for second opinion breast imaging review that included breast and/or axillary ultrasound.

Patient diagnosis on presentation prior to second opinion imaging review	Number of patients (% of total 400)
Invasive carcinoma	199 (50%)
Ductal carcinoma in situ	32 (8%)
High-risk lesion (FEA, ADH, ALH, LCIS)	4 (1%)
Suspicious finding (BI-RADS 4/5)	89 (22%)
Probably benign finding (BI-RADS 3)	36 (9%)
Negative or benign finding (BI-RADS 1 or 2)	34 (7%)
Need additional imaging evaluation (BI-RADS 0)	6 (2%)

FEA: focal epithelial atypia, ADH: atypical ductal hyperplasia, ALH: atypical lobular hyperplasia, and LCIS: lobular carcinoma in situ.

**Table 2:** Analyzed variables for association with ultrasound review discrepancy.

	Patients with US discrepancy/total (%)	*P* value
Dense breasts	68/187 (36%)	0.0001^*∗*^
Not dense breasts	40/213 (19%)	
No prior imaging	20/89 (22%)	0.343
Prior imaging available	88/311 (28%)	
Presenting with malignancy	34/231 (15%)	<0.00001^*∗*^
No malignancy diagnosis at presentation	74/169 (44%)	

^*∗*^Statistically significant.

## Data Availability

The electronic patient record data used to support the findings of this study are restricted by the Moffitt Institutional Review Board in order to protect patient privacy. Data are available from the corresponding author, Robert Jared Weinfurtner, MD (robert.weinfurtner@moffitt.org), for researchers who meet the criteria for access to confidential data.
